# Health care utilization for patients with stroke: a 3-year cross-sectional study of China’s two urban health insurance schemes across four cities

**DOI:** 10.1186/s12889-021-10456-x

**Published:** 2021-03-18

**Authors:** Yong Yang, Stephen Nicholas, Shuo Li, Zhengwei Huang, Xiaoping Chen, Yong Ma, Xuefeng Shi

**Affiliations:** 1 School of Management, Beijing University of Chinese Medicine, Beijing, China; 2 Australian National Institute of Management and Commerce, Sydney, New South Wales Australia; 3 Guangdong Institute for International Strategies, Guangdong University of Foreign Studies, Guangzhou, China; 4 School of Economics and School of Management, Tianjin Normal University, Tianjin, China; 5 Newcastle Business School, University of Newcastle, Newcastle, Callaghan Australia; 6 China Health Insurance Research Association, Beijing, China; 7 National Institute of Traditional Chinese Medicine Strategy and Development, Beijing University of Chinese Medicine, Beijing, China

**Keywords:** Stroke, Healthcare utilization, Health insurance, Medical costs

## Abstract

**Background:**

Stroke is a devastating disease and a major cause of death and disability in China. While existing studies focused mainly on differences in stroke patients’ health care utilization by insurance type, this study assesses whether health utilization and medical costs differed by insurance type across four cities in China.

**Methods:**

A 5% random sample from the 2014–2016 China Urban Employees’ Basic Medical Insurance (UEBMI) and Urban Residents’ Basic Medical Insurance (URBMI) claims data were collected across four cities, Beijing, Shanghai, Tianjin, and Chongqing. Descriptive statistics and ordinary least squares regression were employed to analyze the data.

**Results:**

We found that differences in healthcare utilization and inpatient and outpatient medical expenses varied more by city-specific insurance type than they did between the UEBMI and URBMI schemes. For example, the median UEBMI medical outpatient costs in Beijing (RMB500.2) were significantly higher than UEBMI patients in Shanghai (RMB260.8), Tianjin (RMB240.8), and Chongqing (RMB293.0), and Beijing URBMI patients had significantly higher outpatient medical costs (RMB356.9) than URBMI patients in Shanghai (RMB233.4) and Chongqing (RMB211.0), which were significantly higher than Tianjin (RMB156.2). Patients in Chongqing had 66.4% (95% CI: − 0.672, − 0.649) fewer outpatient visits, 13.0% (95% CI: − 0.144, − 0.115) fewer inpatient visits, and 34.2% (95% CI: − 0.366, − 0.318) shorter length of stay than patients in Beijing. The divergence of average length of stay and out-of-pocket (OOP) expenses by insurance type was also greater between cities than the UEMBI-URBMI mean difference.

**Conclusions:**

Significant city-specific differences in stroke patients’ healthcare utilization and medical costs reflected inequalities in health care access. The fragmented social health insurance schemes in China should be consolidated to provide patients in different cities equal financial protection and benefit packages and to improve the equity of stroke patient access to health care.

## Introduction

As the second most common cause of death, and one of most significant causes of disability, stroke is a leading global public health issue [1, 2]. Compared to all other countries, China has the highest incidence of stroke [3]. In China, the incidence of first-ever stroke in adults grew at an annual rate of 8.3%, with the prevalence of ischemic stroke and hemorrhagic stroke growing significantly [4], even as the age-standardized mortality for ischemic stroke declined by 22.1% and for hemorrhagic stroke by 37.0% between 2005 and 2016 [5]. Increasing stroke incidence, and higher survival rates, imposed a significant financial burden on China’s health care system [6–8], estimated to be about $US6 billion in 2011 [9]. Stroke was also one of the major causes of financial burden on stroke families, and imposed stresses on families through the burden of caring for family stroke victims [10–12].

There are many factors influencing the costs of stroke treatment, such as patients’ sex and age, family financial status and insurance type [13, 14]. In terms of health insurance in China, there were two major urban health insurance schemes, the Urban Employee Basic Medical Insurance (UEBMI) for those in work and the Urban Residents Basic Medical Insurance (URBMI) for the unemployed, retirees, children, and students. Since the two insurance schemes were separately managed at 333 different prefecture (municipal)-level cities in 2012, there were approximately 333 different UEBMI and 333 different URBMI schemes in China [15]. First, the general UEBMI and URBMI schemes varied by the sources of funding, benefit packages, service coverage and financial protection [15–18]. The UEBMI scheme usually had a larger service coverage, better benefit packages and superior financial protection than URMBI, with UEBMI having different rules on reimbursement rates specified in the benefit schedule. The health schemes paid the hospital some share of the hospital expenses, with UEBMI more generous than URBMI. The reimbursement rates of inpatient services in UEBMI and URBMI were usually higher than outpatient reimbursements in both schemes. Second, there were also significant differences in health care access and benefits in the 333 different cities for the UEBMI and URBMI insured.

Depending on the coverage, benefit schedule and reimbursements, a patient’s health insurance scheme impacted the financial burden of stroke on families [19]. Insurance scheme provided a safety net from financial loss, depending on the scope and incentives or disincentives for patients to consume health services [20]. Previous studies have identified that patients covered by UEBMI had higher medical costs than those covered by URBMI [7, 21]. Using the urban health insurance claims database of Guangzhou city, Zhang et al. [19] found that UEBMI inpatients had higher hospital expenses than URBMI inpatients suffering four different subtypes of stroke. Conducting a cross-sectional study, Zhu et al. [7] also found that UEBMI patients had higher medical costs than URBMI patients. What complicates the assessment of stroke health utilization and medical costs in China is that not only are the general conditions in UEBMI more favorable than those in URBMI, but that there are also differences in the terms of UEBMI and URBMI across cities. Existing studies focused only on the general disparities in UEBMI and URBMI, but not whether UEBMI and URBMI health care utilization and medical cost also varied significantly across different cities and prefectures.

To investigate how health care utilization and medical cost varied across different cities and prefectures for each scheme, we collected 3-year health insurance claims data on the average length of hospital stay (ALOS), number of hospital visits and costs of stroke for each type of insurance in each of four province-level cities, Beijing, Shanghai, Tianjin and Chongqing. Differences in utilization rates (ALOS and number of hospital visits) and medical cost (the costs of stroke care) across the four cites reveal how city-level differences in health schemes shaped health care access.

## Materials and methods

### Data source

Data were collected from the China Health Insurance Research Association’s UEBMI and URBMI patient claims, which was a 5% random sample of insurance beneficiaries in 31 provinces in China. We chose patients’ data from four cities: Beijing, Shanghai, Tianjin and Chongqing from the year 2014 to 2016. The four cities are the only provincial level cities administered directly by the Central People’s Government, which display different economic, political, cultural and geographic characteristics between themselves and between China’s other 329 cities. In 2018, the four cities displayed different urban disposable per capita incomes (Beijing RMB62361.2, Shanghai RMB64182.6, Tianjin RMB39506.1 and Chongqing RMB26385.8), which also tended to be higher than the average per capita income for all provinces (RMB 28228) [22]. Since the UEBMI and URBMI are pooled at municipal levels, we can infer differences between all 333 cities in China by evaluating differences between these four cities. The claim data comprised medical information on each stroke patient, including direct stroke medical cost, health insurance name, primary diagnosis (classified according to International Classification of Diseases, 10th edition (ICD-10)), city, out-of-pocket (OOP) expenses and length of hospital stay.

### Study design and patient selection

This is a 3-year cross-sectional study designed to evaluate the health service utilization of stroke patients according to the different cities’ health insurance schemes. Between 2014 and 2016, we obtained all related information on patients whose primary diagnosis was stroke according to ICD-10 (ischemic stroke (I63), hemorrhagic stroke (I60, I61, I62, G45) and other types of stroke (I64, I67-I69, 7, 10) [19]. The final sample included 69,054 patients and 203,697 admissions, including 52,412 patients who were insured by UEBMI and 16,642 patients who were covered by URBMI. Each patient usually had several outpatient or inpatient records in the database. A retrospective, prevalence-based approach was adopted to identify their health care utilization.

### The main variables

For patients from the four cities, the health insurance claims database contained information on the direct total medical cost of stroke, compensation fee (share of total expenses paid by insurance), within-insurance OOP expenses (share of total medical expenses paid by the patient, but listed in the insurance benefit list), outside-insurance OOP expenses (share of total medical expenses paid by the patient, but not listed in the insurance benefit list), average length of stay, number of hospitalizations and outpatient visits. Table 1 displays definitions of four key cost indicators in our study. The median number of outpatient visits and inpatient visits and the ALOS were used to measure outpatient and inpatient health care utilization. The costs of medical care, comprising total medical expenses, within-insurance OOP expenses, and outside-insurance OOP expenses, were the medical cost variables. The Basic Medical Insurance Reimbursement Directory specified health services covered by the two urban insurance schemes and the reimbursement rates for these services. The Directory’s benefits and reimbursement rates varied between UEBMI and URBMI and varied across cities. The control variables included sex (male and female), age, stroke subtype (ischemic stroke, hemorrhagic stroke, and other type of stroke) and year (2014–2016).

**Table 1:** Four cost indicators

Indicators	Comment
Total medical costs	All medical costs generated in one visit, including cost of drugs, medical checks and treatment.
Within- insurance OOP expenses	The proportion of total medical costs specified in the directory, but not reimbursed by the health insurance scheme, including drugs, medical checks and treatment expenses. (paid by patients)
Outside-insurance OOP expenses	Health care expenses not listed in the directory and not compensated by the health insurance scheme, including drugs, medical checks and treatment expenses. (paid by patients)
Reimbursement rate	Proportion of compensation fee (share of total expenses paid by insurance scheme) in total medical cost.

### Statistical analysis

Descriptive statistics were employed to analyze the demographic information. Using median (interquartile range), we calculated outpatient and inpatient healthcare utilization, total medical costs and OOP expenses by insurance type and city. Since the number of outpatient/inpatient visits and ALOS had a non-normal distribution, we used the Kruskal–Wallis test to evaluate the differences in patients’ health care utilization by inter-city UEBMI and URBMI. To identify the influence of insurance type and cities on health care utilization, OLS regression was adopted. To deal with the skewness of data, we converted the number of outpatient visits, number of inpatient visits and ALOS to their natural logarithm. Regression coefficients are routinely interpreted in terms of percent change, and all the regression results (β) have been transformed using the formula, *Coefficient* = *e*^β^ − 1. Statistical analyses were conducted using STATA version 14.0 (Stata Corp LP, College Station, TX) and statistical significance was considered as *p* = 0.05.

## Results

### Patient characteristics

As shown in Table 2, there were 69,054 patients in our sample: 43016 outpatients and 26,038 inpatients; 52,412 were UEBMI patients, with an average age of 66.5 and 16,642 were URBMI patients with an average age of 67.7 years. In the UEBMI group, 71.4% were outpatients and 28.6% were inpatients while 33.6% were outpatients and 66.4% inpatients in URBMI group. Beijing had the largest proportion of UEBMI patients (48.1%) and the largest proportion of outpatients (54.0%); Chongqing had the most inpatients (42.9%) and the highest proportion of URBMI patients (66.3%). Reflecting the broad differences between the UEBMI and URBMI scheme conditions, 15.9 days ALOS for UEBMI was significantly longer than 10.6 ALOS for URBMI. By year, 19,113 patients (27.68%)were from 2014; 29,876 patients (43.26%) were from the 2015; and 20,065 patients (29.06%) were from 2016.

**Table 2:** Socio-demographic characteristics of stroke patients by types of health insurance, 2014–2016

Characteristics	Overall	UEBMI	URBMI	Outpatient	Inpatient
Insurance type					
UEBMI	52,412 (75.9)	–	–	37,422 (87.0)	14,990 (57.6)
URBMI	16,642 (24.1)	–	–	5594 (13.0)	11,048 (42.4)
Visit category					
Outpatient	43,016 (62.3)	37,422 (71.4)	5594 (33.6)	–	–
Inpatient	26,038 (37.7)	14,990 (28.6)	11,048 (66.4)	–	–
Age (years)	66.8 ± 12.2	66.5 ± 12.2	67.7 ± 12.0	65.5 ± 12.0	69.1 ± 12.1
ALOS (days)	13.7 ± 16.8	15.9 ± 19.5	10.6 ± 11.7	–	13.7 ± 16.8
Cities					
Beijing	26,132 (38.2)	25,211 (48.1)	921 (5.5)	23,231 (54.0)	2901 (11.1)
Shanghai	4262 (6.2)	4452 (8.5)	465 (2.8)	655 (1.5)	4262 (16.4)
Tianjin	20,056 (29.3)	15,828 (30.2)	4228 (25.4)	12,362 (28.7)	7694 (29.5)
Chongqing	17,949 (26.2)	6921 (13.2)	11,028 (66.3)	6768 (15.7)	11,181 (42.9)
Year					
2014	19,113 (27.68)	16,021 (30.57)	3092 (18.58)	13,129 (30.52)	5984 (22.98)
2015	29,876 (43.26)	22,465 (42.86)	7411 (44.53)	19,549 (45.45)	10,327 (39.66)
2016	20,065 (29.06)	13,926 (26.57)	6139 (36.89)	10,338 (24.03)	9727 (37.36)

n (%) for categorical variables and mean ± standard deviation for continuous variables

*UEBMI* Urban Employee Basic Medical Insurance scheme, *URBMI* Urban Resident Basic Medical Insurance scheme

### Health care utilization and medical cost of outpatients in four cities

Our results in Table 3 reveal significant differences between UEBMI and URBMI in outpatient healthcare utilization and medical costs by stroke patients. UEBMI outpatients had significantly higher total medical cost, but fewer within insurance OOP expense, than URBMI outpatients. The differences were mostly due to the broad differences in insurance reimbursement rates between the UEBMI and URBMI schemes.

**Table 3:** Outpatient health service utilization per patient by cities

	Beijing		Shanghai		Tianjin		Chongqing		***P***-value
	UEBMI	URBMI	UEBMI	URBMI	UEBMI	URBMI	UEBMI	URBMI	
Median total medical cost (RMB)	500.2	356.9	260.8	233.4	240.8	156.2	293.0	211.0	< 0.001
IQR	[267.0,885.7]	[180.0,686.7]	[180.0,399.9]	[142.4,348.4]	[143.9,447.3]	[109.0,261.2]	[91.3,585.9]	[103.5,407.7]	
Median within- insurance OOP expenses (RMB)	61.8	151.2	70.0	112.6	90.9	93.7	0.1	11.2	< 0.001
IQR	[28.8,133.4]	[38.2,356.7]	[40.6,136.2]	[64.4,150.0]	[39.0,200.1]	[62.9,170.3]	[0.0,34.9]	[4.6,24.2]	
Median outside insurance OOP expenses (RMB)	0.0	0.0	0.0	0.0	0.0	0.4	0.0	0.0	< 0.001
IQR	[0.0,23.6]	[0.0,0.0]	[0.0,0.0]	[0.0,0.0]	[0.0,1.0]	[0.0,1.0]	[0.0,0.0]	[0.0,4.2]	
Reimbursement rate (%)	88.9	50.0	75.9	51.1	62.5	0.0	97.5	89.9	< 0.001
IQR	[82.4,88.9]	[0.0,50.0]	[69.8,80.0]	[50.0,60.0]	[50.7,75.0]	[0.0,50.0]	[88.3,100.0]	[85.8,94.8]	
Median number of visits	3.0	3.0	3.0	3.0	2.0	2.0	1.0	1.0	< 0.001
IQR	[2.0,7.0]	[1.0,5.0]	[1.0,6.0]	[1.0,5.25]	[1.0,4.0]	[1.0,4.0]	[1.0,2.0]	[1.0,1.0]	

*P*-values were based on the Kruskal-Wallis test

*UEBMI* Urban Employee Basic Medical Insurance scheme, *URBMI* Urban Resident Basic Medical Insurance scheme, *SD* standard deviation, *OOP* out-of-pocket, *IQR* interquartile range

In addition, there were also significant intercity UEBMI and URBMI differences in outpatient health care utilization and medical costs. For outpatients, the UEBMI Beijing median (interquartile range, IQR) annual visits (3.0 [IQR = 2.0,7.0]) and Shanghai median annual visits (3.0 [IQR = 1.0,6.0]) were significantly greater than UEBMI Tianjin (2.0 [IQR = 1.0,4.0]) or Chongqing (1.0 [IQR = 1.0,2.0]) median annual visits. URBMI outpatients in Chongqing had significantly fewer median annual visits (1.0 [IQR = 1.0,1.0]), than Shanghai (3.0 [IQR = 1.0,5.25]), Beijing (3.0 [IQR = 1.0,5.0]) and Tianjin (2.0 [IQR = 1.0,4.0]). Median medical cost of the UEBMI group in Beijing (RMB500.2 [IQR = 267.0,885.7]) was higher than the UEBMI group in Shanghai (RMB260.8 [IQR = 180.0,399.9]), Tianjin (RMB240.8 [IQR = 143.9,447.3]) and Chongqing (RMB293.0 [IQR = 91.3,585.9]). The median within-insurance OOP expenses of the UEBMI group in Beijing (RMB61.8 [IQR = 28.8,133.4]) was lower than the UEBMI group in Shanghai (RMB70.0 [IQR = 40.6,136.2]) and Tianjin (RMB90.9 [IQR = 39.0,200.1]), but higher than Chongqing (RMB0.1 [IQR = 0.0,34.9]). The reimbursement rate of the UEBMI scheme in Chongqing was higher than the UEBMI or URBMI group in Beijing, Tianjin and Shanghai.

### Health care utilization and medical cost of inpatients in four cities

Reflecting the Directory benefits of the insurance schemes and inpatient care preferences, Table 4 reports that there were significant differences between UEBMI and URBMI in inpatient health care utilization and medical costs. While the UEBMI group had a similar number of inpatient visits as the URBMI group across the four cities, the UEBMI group had significantly longer ALOS, and significantly higher medical cost, than the URBMI group in each of the four cities. Except in Chongqing, UEBMI patients had a significantly overall lower within-insurance OOP expenses than URBMI patients, and except for Beijing, UEBMI patients had significantly overall higher outside-insurance OOP expenses.

**Table 4:** Inpatient health service utilization per patient by cities

	Beijing		Shanghai		Tianjin		Chongqing		***P***-value
	UEBMI	URBMI	UEBMI	URBMI	UEBMI	URBMI	UEBMI	URBMI	
Median total medical cost (RMB)	16,275.8	14,473.4	13,930.0	10,606.1	12,098.4	6960.7	8061.5	5673.3	*P* < 0.001
IQR	[11,287.0,23,755.1]	[9317.5,22861.7]	[9649.4,20520.2]	[6025.4,18239.7]	[7472.1,18801.5]	[3354.7,12289.4]	[4849.3,14928.4]	[3403.3,10467.3]	
Median within- insurance OOP expenses (RMB)	1719.4	4889.1	1822.3	2001.1	2251.5	2761.9	623.5	352.1	*P* < 0.001
IQR	[1076.5,2225.9]	[3381.2,6747.3]	[1119.1,2808.0]	[771.1,3482.8]	[1333.1,3206.2]	[1249.7,5010.4]	[330.0,1244.9]	[168.8,682.7]	
Median outside insurance OOP expenses (RMB)	28.3	179.2	210.0	98.0	1068.6	503.1	433.0	215.7	*P* < 0.001
IQR	[0.0,204.5]	[60.2,681.3]	[18.0,445.7]	[0.0,296.4]	[338.3,2841.7]	[84.0,1671.3]	[158.6,1105.3]	[77.6,564.5]	
Reimbursement rate (%)	89.4	64.9	87.5	79.6	80.4	55.3	91.4	89.5	
IQR	[84.9,93.6]	[61.2,67.2]	[82.7,90.6]	[79.1,89.0]	[75.2,84.9]	[47.7,65.6]	[88.8,93.6]	[86.8,92.6]	
Median number of visits	1.0	1.0	1.0	1.0	1.0	1.0	1.0	1.0	*P* < 0.001
IQR	[1.0,1.0]	[1.0,1.0]	[1.0,2.0]	[1.0,1.0]	[1.0,1.0]	[1.0,1.0]	[1.0,1.0]	[1.0,1.0]	
Average LOS (days)	14.0	13.0	15.0	13.0	14.0	12.0	11.0	8.0	*P* < 0.001
IQR	[10.0,21.0]	[9.0,21.0]	[10.0,35.0]	[9.0,20.0]	[10.0,17.0]	[7.0,14.0]	[7.0,17.0]	[5.0,13.0]	

*P*-values were based on the Kruskal-Wallis test

*UEBMI* Urban Employee Basic Medical Insurance scheme, *URBMI* Urban Resident Basic Medical Insurance scheme, *SD* standard deviation, *OOP* out-of-pocket, *LOS* length of stay, *IQR* interquartile range

Table 4 also indicates that there were significant differences between intercity UEBMI and URBMI schemes in inpatient health care utilization and medical costs. Inpatients with UEBMI in Shanghai had significantly longer annual median ALOS days (15.0 [IQR = 10.0,35.0]) than UEBMI inpatients in Beijing (14.0 [IQR = 10.0,21.0]), Tianjin (14.0 [IQR = 10.0,17.0]) and Chongqing (11.0 [IQR = 7.0,17.0]). URBMI inpatients in Shanghai (13.0 [IQR = 9.0,20.0]) and Beijing (13.0 [IQR = 9.0,21.0]) had significantly longer annual median ALOS than URBMI inpatients in Tianjin (12.0 [7.0,14.0]) and Chongqing (8.0 [5.0,13.0]). Inpatients with UEBMI in Beijing (RMB16275.8 [IQR = 11,287.0,23,755.1]) had significantly higher median medical costs than UEBMI patients in Shanghai (RMB13930.0 [IQR = 9649.4,20520.2]), Tianjin (RMB12098.4 [IQR = 7472.1,18801.5] and Chongqing (RMB8061.5[IQR = 4849.3,14928.4]). URBMI inpatients in Beijing (RMB14473.4 [IQR = 9317.5,22861.7]) also had significantly higher medical costs than URBMI inpatients in Shanghai (RMB10606.1 [IQR = 6025.4,18239.7]), Tianjin (RMB6960.7 [IQR = 3354.7,12289.4]) and Chongqing (RMB5673.3 [IQR = 3403.3,10467.3]). Table 4 also shows the significant disparities in the city-by-city UEMBI and URBMI within-insurance OOP expenses, without-insurance OOP expenses and the reimbursement rate. Importantly, from the perspective of the deviation of median values, the UEBMI and URBMI inpatient medical costs and healthcare utilization differences across the cities were greater than the differences between the UEBMI and URBMI averages.

### OLS regression

Table 5 shows the influence of insurance type and city on health services utilization. We found that insurance type and city were significantly associated with patients’ health care utilization. First, outpatient and inpatient visits and ALOS varied significantly by UEBMI-URBMI schemes. URBMI patients with stroke had 12.2% (95% CI: − 0.144, − 0.100)/4.1% (95% CI: − 0.050, − 0.032) fewer outpatient/inpatient visits than UEBMI patients, and UEBMI patients had 27.0% (95% CI: − 0.288, − 0.251) longer ALOS than URBMI patients. Second, health care utilization significantly varied by city: patients in Tianjin had 28.0% (95% CI: − 0.294, − 0.267) fewer outpatient visits than Beijing patients. The gap between patients in Chongqing (66.4, 95% CI: − 0.672, − 0.649) and patients in Beijing was even greater for the number of outpatient visits. Patients in Shanghai had 12.2% (95% CI: 0.097, 0.147) more inpatient visits and 27.8% (95% CI: 0.223, 0.335) longer ALOS than patients in Beijing, while patients in Chongqing had 13.0% (95% CI: − 0.144, − 0.115) fewer inpatient visits and 34.2% (95% CI: − 0.366, − 0.318) shorter ALOS than patients in Beijing. The coefficients for inter-city differences in health care utilization for stroke patients were greater than the differences in coefficients between UEBMI and URBMI.

**Table 5:** The impact of insurance type and city on patients’ health care utilization

Characteristics	Outpatient visit		Inpatient visit		ALOS	
	Coef.	95%CI	Coef.	95%CI	Coef.	95%CI
Insurance type (Ref: UEBMI)						
URBMI	−0.122^***^	[−0.144, −0.100]	−0.041^*^	[−0.050, −0.032]	−0.270 *	[−0.288, −0.251]
Cities (Ref: Beijing)						
Shanghai	−0.054	[− 0.121, 0.018]	0.122^***^	[0.097, 0.147]	0.278 ^***^	[0.223, 0.335]
Tianjin	−0.280^***^	[−0.294, − 0.267]	0.025^***^	[0.007, 0.043]	− 0.155 ^***^	[− 0.184, − 0.125]
Chongqing	−0.664^***^	[− 0.672, − 0.649]	−0.130^***^	[− 0.144, − 0.115]	−0.342 ^***^	[− 0.366, − 0.318]
**R**^**2**^ **(adjusted)**	0.189		0.100		0.130	

All models were adjusted for gender, age, stroke type and year; All coefficients and 95% CI were transformed using formula Coef. = e^β^ − 1

*ALOS* average length of stay

^***^P < 0.001, ^**^P < 0.01, ^*^P < 0.05

## Discussion

Consistent with findings in previous studies [21, 23–25], we found that stroke patients with UEBMI consumed more health services and incurred higher medical costs than those covered by URBMI. Our most important finding was that the utilization rate and medical cost of UEMBI and URMBI differed significantly across different cities, and these intercity differences in health care utilization were greater than the differences between UEBMI and URBMI.

There are several possible explanations accounting for UEBMI patients across the four cities utilizing different health care than URBMI patients. Since socioeconomic status and education attainment have been found to be important influential factors in pre-hospital delay, which will affect the ALOS for ischemic stroke inpatients [26], we speculate that patients covered by UEBMI had higher levels of education and socioeconomic status than URBMI members, as well as paying more attention to their personal health [20]. Consequently, UEBMI members sought medical treatment at higher level hospitals and were more willing to consume additional health services that were not covered by health insurance than URBMI patients [16, 27]. Second, different therapeutic schedules could be adopted by doctors according to patients’ insurance status, and the UEBMI insurance scheme benefit schedule was more generous, and offered a higher reimbursement rate, than URBMI [14, 28]. Importantly, UEMBI and URBMI benefit schedules varied across cities. This meant UEMBI patients in different cities enjoyed more generous benefits, a higher reimbursement rate for services, higher annual reimbursement ceiling and more comprehensive service coverage, both because they were in the UEMBI scheme and they were in a city-specific UEMBI scheme. Two additional behaviors potentially follow. UEBMI members demanded more hospital services than they needed and medical staff supplied more hospital services than required [29]. Since URBMI provided weaker financial protection from hospital expenses than UEBMI, URBMI members had an incentive to curtail their consumption of more health services and doctors were disincentivized in providing excess services [27]. Our key finding is that these different provisions on coverage, benefits and reimbursements were city-specific, where local differences in the UEBMI and URBMI behavior were magnified.

Our UEBMI and URBMI findings share several similarities with previous studies [24, 25]. An empirical study conducted by Luo et al. [30] reported that patients with end-stake malignant tumors covered by UEBMI utilized more health services than those covered by URBMI. Xu et al. [31] used 10 years (2005–2014) of hospital electronic health records to measure the utilization of mental health inpatient services. They found that patients with UEBMI had higher hospitalization cost, OOP costs, reimbursement ratio, greater number of inpatient visits, and much longer ALOS. Wang et al. [23] reported that middle-aged and elderly adults covered by UEBMI had a larger number of outpatient visits and longer ALOS than those covered by URBMI. Meanwhile, the UEBMI group had higher outpatient OOP costs and higher inpatient OOP costs, but much fewer total average OOP costs.

In terms of city-specific differences in health care utilization and medical costs between UEBMI and URBMI, there are several possible explanations. First, different economic level cities had different benefit packages and funding standards per capita, which encouraged divergent health care utilization [32, 33]. While all four cities had high per capita incomes, Beijing had the highest UEBMI revenue per capita, followed by Shanghai, Tianjin, and Chongqing in 2018 [34], with Beijing and Shanghai’s health care utilization greater on average than Tianjin or Chongqing. We expect variations in income per capita across the 333 cities in China to result in different benefit schemes, with significant differences in health care utilization.

Second, the prevalence of stroke differed between cities. A higher stroke prevalence contributed to a higher consumption of health services [35]. A study conducted by the China Stoke Data Center reported that from 2012 to 2016, Beijing had the highest prevalence of stroke among these four cities, and stroke prevalence in Chongqing was the lowest [36]. The higher health care utilization in Beijing compared to Chongqing is consistent with different stroke prevalence rates. In addition, as one of the major potentially modifiable risk factors and comorbidities for stroke, hypertension prevalence also differed between cities. Comorbidities significantly increased the utilization of health services in patients with stroke [37]. One study reported that Beijing had the highest prevalence of hypertension of these cities, followed by Tianjin, Shanghai, and Chongqing [38]. These intercity risk factors and comorbidities were likely multiplied across the 333 cities in China.

Third, there were disparities in the social development and economy between the four cities, with the city populations displaying different socioeconomic status. Low socioeconomic status patients had a higher risk of stroke hospitalization and case fatality than those with a high socioeconomic status [39]. Socioeconomic status differences varied even more significantly across the 333 cities than the four cities in our study. Fourth, there were disparities in the charging standard across hospitals in the four cities. According to the guidelines from the national government and implementation plans from local governments, the prices for basic medical services provided by public hospitals were formulated to government guidance prices [40]. But, there is evidence that the use of health services was strongly linked to price [41]. Patients respond to price in two ways: by changing the frequency of service consumption or changing the quality of care to reduce per visit costs [41]. These behaviors would be amplified across the 333 cities in China with different UEBMI-URBMI schemes.

To place our findings in an international comparative context, other studies also found that patients in different cities displayed differences in health care utilization. Hagman [42] found that Finnish patients with a higher glaucoma stage in different districts utilized a different degree of health resources and had differential treatment expenditures. A study in India revealed a wide variation in health care utilization for epileptic sufferers from six Indian cities [43]. In Mexico, a study reported that patients covered by insurance called Seguro Popular de Salud (SPS) in larger cities had significantly fewer OOP expense than their uninsured counterparts, but possibly because of the limited access to health resources, no effect was found among SPS-insured households living in smaller cities [44]. Wang et al. [45] reported that there were wide regional variations of health care utilization and expenditure for patients with type 2 diabetes between Beijing, Guangzhou, Shanghai, and Chengdu.

This study has several limitations. First, extrapolation from our findings from the four cities to the healthcare utilization situation of all patients with stroke in China should be undertaken cautiously because our claims database was restricted to the urban population. We are, however, confident that the intercity differences for the four cities applies across all Chinese cities. Second, when assessing the influence of insurance type and city on healthcare utilization, potential factors such as clinical severity of the disease, comorbidities, personal income levels and education attainment, which could have impacted healthcare utilization and should be controlled, were omitted due to the absence of such data in the claim dataset. Future studies should collect these data. Third, we calculated outpatient and inpatient medical costs for patients with stroke, while indirect costs due to family members’ informal care and loss of productivity were not assessed because our dataset did not include this information. Future studies should consider these indirect costs.

## Conclusion

This study found that healthcare utilization and medical costs of patients with stroke varied by insurance type and by city-specific insurance type across four large Chinese cities, Beijing, Shanghai, Tianjin and Chongqing. The differences across the four cities were greater than the differences between UEBMI and URBMI. Our results call for national guidelines and action plans to consolidate China’s fragmented social health insurance schemes to improve equality in access to health care. Both funding level and benefit packages should be standardized across cities. We suggest that the integration of China’s social health insurance schemes at the national level would reduce inequities in benefit packages and attenuate intercity differences in healthcare utilization.

## Data Availability

As the data is not publicly available, permission to access the data was obtained from China Health Insurance Research Association, and can be made available on reasonable request from the corresponding author with permission from China Health Insurance Research Association.

## Abbreviations

UEBMI: Urban Employees’ Basic Medical Insurance
URBMI: Urban Residents’ Basic Medical Insurance
ALOS: Average length of stay
OOP: Out-of-pocket
IQR: Interquartile range
